# Neuroinflammation: A Critical Factor in Neurodegenerative Disorders

**DOI:** 10.7759/cureus.62310

**Published:** 2024-06-13

**Authors:** Zahieh Suleiman Khoury, Fatima Sohail, Jada Wang, Moises Mendoza, Mohammed Raake, Mohammed Tahoor Silat, Manoj Reddy Bathinapatta, Amirali Sadeghzadegan, Patel Meghana, Janisha Paul

**Affiliations:** 1 Department of Medicine, Los Andes University, Mérida, VEN; 2 Department of Medicine, Tulane University School of Public Health and Tropical Medicine, New Orleans, USA; 3 Department of Medicine, St. George’s University, Brooklyn, USA; 4 Department of Health Sciences, Universidad Centroccidental Lisandro Alvarado, Barquisimeto, VEN; 5 Department of Medicine, Annamalai University, Chennai, IND; 6 Department of Medicine, Dow International Medical College, Karachi, PAK; 7 Department of Medicine, Ivano-Frankivsk National Medical University, Ivano, UKR; 8 Department of General Practice, Marmara University School of Medicine, Istanbul, TUR; 9 Department of Medicine, Ramaiah University of Applied Sciences, Bengaluru, IND; 10 Department of Medicine, Punjab Institute of Medical Sciences, Jalandhar, IND

**Keywords:** therapeutic strategies, alzheimer’s disease, parkinson’s disease, microglia, chemokines, cytokines, astrocytes, neuroinflammation, neurodegenerative disorders

## Abstract

This review offers a comprehensive review of the signals and the paramount role neuroinflammation plays in neurodegenerative diseases such as Alzheimer’s, Parkinson’s, Huntington’s, and amyotrophic lateral sclerosis. The study explores the sophisticated interactions between microglial, astrocytic, and dendritic cells and how neuroinflammation affects long-term neuronal damage and dysfunction. There are specific pathways related to the mentioned inflammatory processes, including Janus kinases/signal transducer and activator of transcriptions, nuclear factor-κB, and mitogen-activated protein kinases pathways. Neuroinflammation is argued to be a double-edged sword, being not only a protective agent that prevents further neuron damage but also the causative factor in more cell injury development. This concept of contrasting inflammation with neuroprotection advocates for the use of therapeutic techniques that seek to modulate neuroinflammatory responses as part of the neurodegeneration treatment. The recent research findings are integrated with the established knowledge to help present a comprehensive image of neuroinflammation’s impact on neurodegenerative diseases and its implications for future therapy.

## Introduction and background

Neurodegenerative disorders involve conditions characterized by the progressive breakdown of nerve cells in the brain and spinal cord, primarily marked by the loss of neurons. Neurons are central to the proper functioning of the human brain, playing a critical role in communication. Most neurons are created in the brain, but they are everywhere in the body. Neurons are not immortal, but the progressive loss of neurons and their structure or function leads to the pathophysiology of several brain disorders and significant health concerns [1]. The most common neurodegenerative disorders include Alzheimer’s, Parkinson’s, epilepsy, stroke, Huntington’s, and amyotrophic lateral sclerosis (ALS). Neurodegeneration occurs when there is a problem with how the brain cells communicate, how they form networks, and when abnormal proteins build up in the brain. Neuroinflammation occurs when parts of the central nervous system (CNS) react to changes in balance regardless of whether the cause is internal or external. Neuroinflammation is a process of infiltration of immune and inflammatory cells of blood origin and tissue injury in the CNS. Due to the increased cell permeability and vasodilation, damage signals produce a response in the CNS to limit or block the reaction or the misfolded proteins that act as initiators [2]. Over time, this permanent activation of CNS protective cells produces a change in cell structures and functions, making a pathway to mutations, furthering the environmental damage, degeneration, and death of cells. When our body’s defenses kick in, they help heal tissue damage or fight off infections. After successfully addressing a threat, the immune system normally returns to a resting state. However, if the underlying cause of inflammation persists, it can become chronic, leading to long-term health problems [3]. Chronic inflammation can release harmful substances that can worsen the disease. Persistent inflammation can be caused by building clumps of proteins and other factors. Special fats called specialized pro-resolving lipid mediators (SPMs) help reduce inflammation and promote tissue healing. If the body does not produce enough SPMs, inflammation can persist and lead to chronic diseases [4].

The initial event in neuroinflammation often involves the alteration of blood-brain barrier (BBB) permeability. This allows immune cells from the bloodstream to infiltrate the brain and activate resident microglial cells, leading to astrocytosis and further inflammatory responses. Microglia and astrocytes are key players in neuroinflammation. Microglia act as the brain’s resident immune cells, continuously monitoring the environment for signs of infection or injury. They become activated in response to pathogen-associated molecular patterns (PAMPs) and damage-associated molecular patterns (DAMPs). Once activated, microglia release pro-inflammatory cytokines such as interleukin (IL)-1β, tumor necrosis factor-alpha (TNF-α), and chemokines like C-C motif chemokine ligand 2 (CCL2) and IL-18. Astrocytes also play a crucial role by maintaining the BBB, supporting neuronal function, and responding to injury through reactive astrogliosis. This process involves the secretion of cytokines and chemokines that propagate the inflammatory response. Oxidative stress is a significant contributor to neuroinflammation. It results from an imbalance between the production of reactive oxygen species (ROS) and the brain’s ability to detoxify these reactive intermediates. Mitochondrial dysfunction, often seen in neurodegenerative diseases, exacerbates oxidative stress and leads to further neuronal damage and inflammatory responses [5].

Moreover, in neurodegeneration, inflammation plays a key role in triggering the activation of immune cells such as microglia and astrocytes. The immune cells, particularly glial cells, have receptors on their surfaces that detect bodily abnormalities. When these receptors are stimulated by certain substances, such as abnormal proteins, viruses, or bacteria, they activate signal pathways to turn on genes. These genes then inform the cell to release molecules that cause inflammation, leading to the problem. Generally, activated glial cells should eliminate the threats and start to reduce the inflammation and clear any harmful substances. Furthermore, the protein clumps and toxic substances released by damaged neurons can worsen inflammation. In some cases, protein clumps can invade the powerhouse of cells, the mitochondria, causing neurons to die directly [6,7].

The general concept of neuroinflammation revolves around the brain’s immune response to disruptions to its normal functioning. Understanding neuroinflammation in the context of neurodegenerative disease is crucial to developing effective treatments, interventions, and early detection of symptoms. In this review, we have investigated the role of various neurodegenerative disorders and their inflammatory processes, molecular and cellular mechanisms, pathological pathways, future directions for treatments, further research, and emerging trends [8].

## Review

Neuroinflammation: basics and mechanisms

Bioactive lipids, including resolvins, protectins, and maresins, actively terminate the inflammatory response. They function by stopping the further recruitment of immune cells, promoting the clearance of debris and apoptotic cells, and enhancing the repair of damaged tissues. Microglial cells can switch from a pro-inflammatory M1 phenotype to an anti-inflammatory M2 phenotype. This switch is crucial for resolving inflammation, as M2 microglia produce anti-inflammatory cytokines such as IL-10 and TGF-β, which help in tissue repair and return to homeostasis. Astrocytes, another type of glial cell, support the resolution of inflammation by producing anti-inflammatory molecules and growth factors that aid in neuronal survival and repair. They also help in re-establishing the integrity of the BBB, which is often compromised during inflammation. The inflammatory process is modulated by a balance of pro-inflammatory and anti-inflammatory cytokines. While pro-inflammatory cytokines such as IL-1β and TNF-α initiate and sustain the inflammatory response, the production of anti-inflammatory cytokines such as IL-10 helps in damping down the inflammatory signals and promoting resolution [9].

Neuroinflammation initiates and continues a series of events when homeostasis is disrupted. The series of events starts with alterations in the permeability of the BBB (Table 1). Then, immune cells from the bloodstream infiltrate the brain, and residential brain microglial cells become activated, along with astrocytosis [10]. Neuroinflammation describes the responses to external and internal disturbances from reactive components within the CNS. Neuroinflammation responds to harmful agents in the nervous system, such as infections or other harmful factors. Various neurological conditions involve both direct and indirect immune-related neuroinflammation. Examples of neurological conditions include trauma, tumors, ischemia, metabolic disorders, toxicity, infections, autoimmune disorders, developmental anomalies, and degenerative diseases. In response to harmful agents, the brain will receive infiltrations from innate and adaptive immune system cells, releasing inflammatory signaling molecules. This can lead to detrimental consequences within the brain tissue, potentially resulting in demyelination or varying degrees of neuronal changes. To find ways to treat or lessen harm to the CNS, we need to learn more about immune-related inflammation in healthy and sick people [11].

**Table 1:** Various neuroinflammation pathways.

Pathway	Description	Reference
Nuclear factor-kappa B (NF-κb)	A critical modulator of inflammatory reactions is nuclear factor-kappa B, controlling the expression of pro-inflammatory cytokines and chemokines	[12]
Janus kinase/Signal transducer and activator of transcriptions (JAKs/STAT)	Signal transducer and activator of transcription and Janus kinase are proteins that mediate the signaling of cytokines and inflammation	[12]
Mitogen-activated protein kinase (MAPK)	Protein kinase pathways, such as JNK, p38, and ERK, control the expression of genes related to inflammation	[12]
Toll-like receptor (TLR)	Triggering innate immune responses and the production of pro-inflammatory cytokines are TLRs, which are receptors that recognize patterns	[12]
NLRP3 inflammasome activation	NLRP3 inflammasome is a multiprotein complex involved in producing IL-18 and IL-1β, both of which are pro-inflammatory cytokines	[13]
PI3K-Akt pathway	Protein kinase B and phosphoinositide 3-kinase signaling pathways regulate cell survival and inflammation	[14]
Notch pathway	The notch pathway can modulate inflammation and immune responses in the central nervous system	[15]
HIF-1α pathway	Hypoxia-inducible Factor 1-alpha can influence inflammation under hypoxia conditions.	[16]
Rho GTPase signaling	Rho family GTPases can regulate cytoskeletal changes and cell migration associated with neuroinflammation	[17]

The BBB consists of an endothelial layer, surrounded by a basal lamina, ensconced by astrocyte endfeet, and accompanied by pericytes. Astrocytes and their connections with neurons form the neurovascular unit. Inflammation represents the body’s physical reaction to a series of immune system processes designed to protect, defend, and promote healing. Various factors can trigger inflammation, and its effects are coordinated by numerous cell types and signaling molecules, leading to both localized and systemic reactions. White blood cells, specifically monocytes, are the leading players in the broader inflammatory response. The lymphoid system is directly innervated by peptidergic, catecholaminergic fibers, and adrenergic allowing the neurological system to form functional linkages with the immune system. Additional neuropeptides and neurotransmitters the immune system interacts with include somatostatin, gastrointestinal peptide, and substance P. The production and secretion of cytokines, including chemokines, are controlled by these. Chemokines serve as central neuromodulators and are responsible for recruiting secondary pro-inflammatory leukocytes [18].

**Figure 1:**
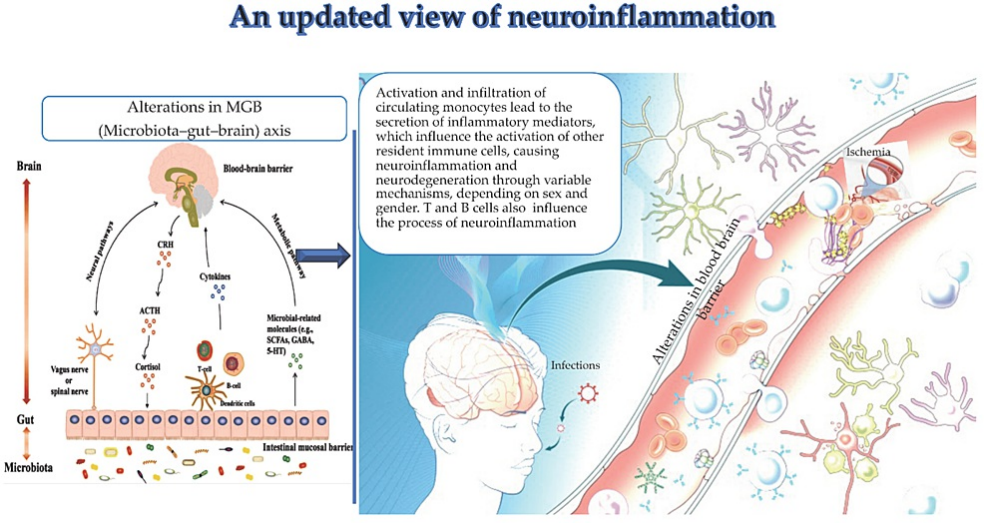
Infections, ischemia, and the microbiota-gut-brain (MGB) axis alterations promote the activation, infiltration, and specific release of inflammatory mediators by circulating monocytes in the brain. The latter produce neuroinflammation and neurodegeneration by inducing the activation of additional local immune cells. Clonal cells have an impact on neuroinflammation. Reproduced under the terms and conditions of the Creative Commons Attribution (CC BY) license (https://creativecommons.org/licenses/by/4.0/) from Balistreri et al. [21]. Copyright © 2023 by the authors. Licensee MDPI, Basel, Switzerland.

Inflammation responses in the CNS rely heavily on microglia and astrocytes. Their activation is divided into two main categories, namely, neurotoxic activation characterized during neuroprotective activation, as shown by M2-phenotype microglia and A2-phenotype astrocytes, and during M1-phenotype microglia and A1-phenotype astrocytes. In the mouse brain, microglial cells comprise 5% to 12% of the total cell population and exhibit various morphologies and shapes. Microglia have three main functions, namely, sensing changes in the environment through sensors; performing essential duties such as migrating to injured sites, reshaping synapses, and maintaining myeline equilibrium; and, lastly, molecular patterns linked with pathogens and damage are included by microglia. Microglia gain the ability to identify DAMPs and PAMPs via the expression of cellular receptors such as nuclear oligomerization domain-like receptors, viral receptors, and toll-like receptors. The production of pro-inflammatory cytokines such as IL-1β, IL-16, and TNF-α as well as chemokines such as CCL2 and IL-18, occurs when microglia are stimulated [19]. This recruitment of immune cells and removal of pathological agents is part of the neuroinflammatory response. It is important to note that while neuroinflammation serves as a neuroprotective mechanism, prolonged neuroinflammation can lead to neurotoxicity and is associated with neurodegenerative conditions. Microglia and astrocytes communicate through various molecular signals, such as cytokines and adenosine triphosphate. Research has shown that activated microglia release cytokines that may generate reactive astrocytes in response to stimuli such as lipopolysaccharides. Microglia may secrete molecular cues that activate reactive astrocytes because of their heightened sensitivity to infections or injury. Stroke and other neuroinflammatory events may include astrocytes and microglia that communicate using a shared molecular language. As the pathology advances, environmental changes promote the transition from M1 to M2. Astrocytes interact with neurons and the circulatory system, integral to the NVU, serving as cells responsible for maintaining neuronal function and the integrity of the BBB [20]. Monocytes and clonotypic immune cells represent key actors of neuroinflammation, as recently underlined (Figure 1).

Neurodegenerative disorders and neuroinflammation

*Alzheimer*’*s Disease and Its Stages*

Deterioration of cognitive abilities is a hallmark of Alzheimer’s disease and daily functioning resulting from the degeneration of brain cells. The primary factor contributing to this disorder is dementia, which starts with a gradual decline in memory function and progresses to an inability to have a conversation and answer appropriately [22]. The development of Alzheimer’s disease symptoms occurs at least 10 years later. Atypical protein deposits in the brain, known as amyloid plaques and tau tangles, mark the beginning of Alzheimer’s disease. When an injury occurs to the memory-forming regions of the brain, the hippocampus and the entorhinal cortex, and more neurons die off, other brain regions also start to atrophy. Some other factors that can trigger progressive loss of cognitive functions are intoxications, infections, abnormalities in the circulatory and pulmonary systems, reduced oxygen supply in the brain, vitamin B12 deficiency, and tumors [23].

**Figure 2:**
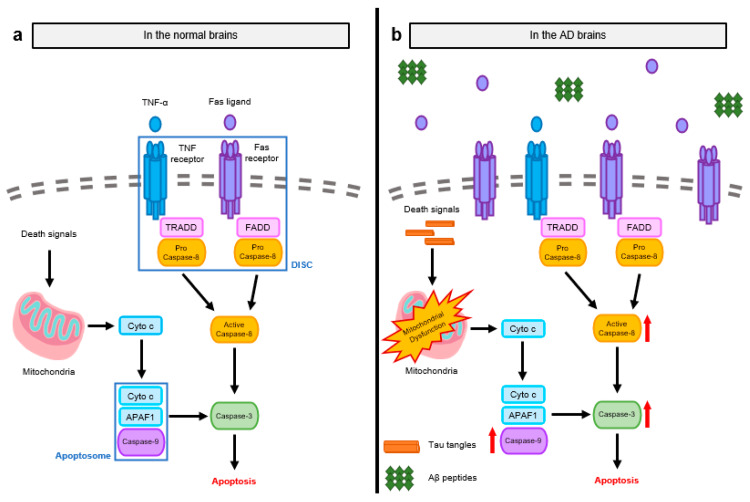
Molecular pathways of apoptosis in the brains of Alzheimer’s disease (AD) patients and normal subjects. (a) There are two primary signaling mechanisms that trigger apoptosis: the intrinsic pathway, which is regulated by mitochondria, and the extrinsic pathway, which is mediated by death receptors (DRs). The activation of pro-apoptotic caspases is required for signaling transmission after the start of apoptosis. Cyto c and APAF1 activate caspase-9, the first caspase in the intrinsic pathway, which causes apoptosomes to form. The oligomerization of DRs and the subsequent recruitment of adaptor proteins, which comprise the DISC, activate caspase-8, the first caspase in the extrinsic route. The brain cells undergo apoptosis as a result of the activation of executioner caspase-3 by activated caspase-9 and -8. (b) Both internal and extrinsic mechanisms are heightened in AD brains due to improper apoptotic regulation. Increased Fas ligands and aberrant protein aggregates cause initial caspases to become more activated. With the high frequency of apoptosis during AD, the activation of early caspases triggers the activation of caspase-3. Reproduced under the terms and conditions of the Creative Commons Attribution (CC BY) license (https://creativecommons.org/licenses/by/4.0/) from Choi et al. [26]. Copyright © 2023 by the authors. Licensee MDPI, Basel, Switzerland.

There are seven stages of Alzheimer’s disease. In the first stage, an individual may experience changes in mood and behavior along with signs of cognitive and functional impairment. Although the individual’s cognition seems unaffected, pathological alterations occur in the brain during stage one. Stage two is subjective memory loss with age-related forgetfulness, which has been very common among individuals over 65. At stage two, patients often start forgetting people’s names, and they usually cannot recall the locations of their most recent possessions. Mild cognitive impairment is the third stage. During this stage, an individual’s capacity to perform executive functions is compromised, and their ability to complete a specific task becomes challenging. Mild dementia and moderate cognitive deterioration constitute stage four. A diminished capacity to perform daily tasks, such as remembering to pay rent and bills or to write down the proper data, is the most prevalent functional deficiency among these people [24]. Some of the symptoms of this stage are forgetting the obvious, such as remembering or recalling the day of the week. Stage five is moderately severe cognitive decline with moderate dementia. In this stage, an individual starts getting characteristic functional changes in basic activities of daily life. Cognitive deterioration occurs during the devastating stage six. When individuals with moderate-to-severe dementia are unable to perform even the most fundamental of everyday tasks, such as dressing themselves or recognizing familiar faces, it is considered a severe form of dementia. Characteristic changes occur throughout this time. Stage seven occurs when a person is terminally ill and exhibiting motor symptoms, communication difficulties, and the need for feeding help (Figure 2) [25].

Role of Neuroinflammation in Alzheimer’s Pathology

Neurofibrillary tangles and aberrant neuritis plaques build up abnormally in Alzheimer’s disease. Extracellular amyloid-beta (Aβ) peptide forms plaques, which are minute lesions that resemble spherical objects [27]. Tau, a protein found in neurons, forms fibrillary intracytoplasmic aggregates termed neurofibrillary tangles, which stabilize axonal microtubules. Tau clumps together to create neurofibrillary tangles, twisted pairs of helical filaments. Tau proteins are ubiquitous in the brain after first appearing in the hippocampus. Compared to plaques, tangles are a more robust indicator of Alzheimer’s disease [28].

Key Inflammatory Markers and Pathways

The presence of abnormally phosphorylated tau protein inside cells and extracellular deposits of Aβ peptide are the clinical characteristics that identify Alzheimer’s disease. Pathophysiological indicators are best retrieved from cerebrospinal fluid. To diagnose Alzheimer’s disease, the most critical biological indicators are total tau, phosphorylated tau isoforms (P-Tau231 and P-Tau 181), and β-amyloid peptide [29]. The existence of diagnostic levels of Aβ42 and phosphorylated tau in the cerebrospinal fluid was verified by researchers who examined plasma and cerebrospinal fluid from 48 individuals with mild cognitive impairment. Additionally, the disease’s biochemical processes have not been identified yet [25].

Neuroinflammation in the main neurodegenerative disorders

Neuroinflammation and pathogenesis in Parkinson’s disease are based on several factors, mainly molecular dysfunctions [30]. One of the main proteins in the CNS, alpha-synuclein, misfolds and aggregates due to the incapacity of the dopaminergic neurons to manage oxidative stress, which leads to the accumulation of alpha-synuclein, gaining a toxic function at the same time. The overexpression of alpha-synuclein increases the levels of oxidative stress and changes the structure of the mitochondria, which decreases the amount of ATP produced and increases the generation of free radicals, followed by the accumulation of alpha-synuclein [31,32].

Every CNS cell plays a role in neuroinflammation via alpha-synuclein. The neuronal alpha-synuclein immediately affects the astrocytes, which causes inflammation and triggers the secretion of IL-6, IL-1γ, ICAM-1, and CXCL10. Alpha-synuclein enhances γ-syn phagocytosis in microglia via FcγR receptors, which, in turn, triggers a cascade of pro-inflammatory events such as the translocation of NFαB p65 to the nucleus and an increase in the production of cytokines. As a result, these processes amplify the loss of neurons and persistent neurodegeneration in Parkinson’s disease. Moreover, lymphocytes recognize misfolded proteins during neuroinflammation, promoting cytotoxicity to DA neurons [33]. In Huntington’s disease, activated microglia and astrocytes perform major inflammatory processes. Depending on the region analyzed, the neuroinflammation factors vary; nevertheless, studies show that the main factors are the accumulation of mHTT; interleukins such as IL-6, IL-8, IL-10; cytokines such as TGF-β and TNF-α; chemokines; the presence of ROS and MMP; and proteins such as clustering [34].

Role of neuroinflammation in neurodegenerative disorders

**Figure 3:**
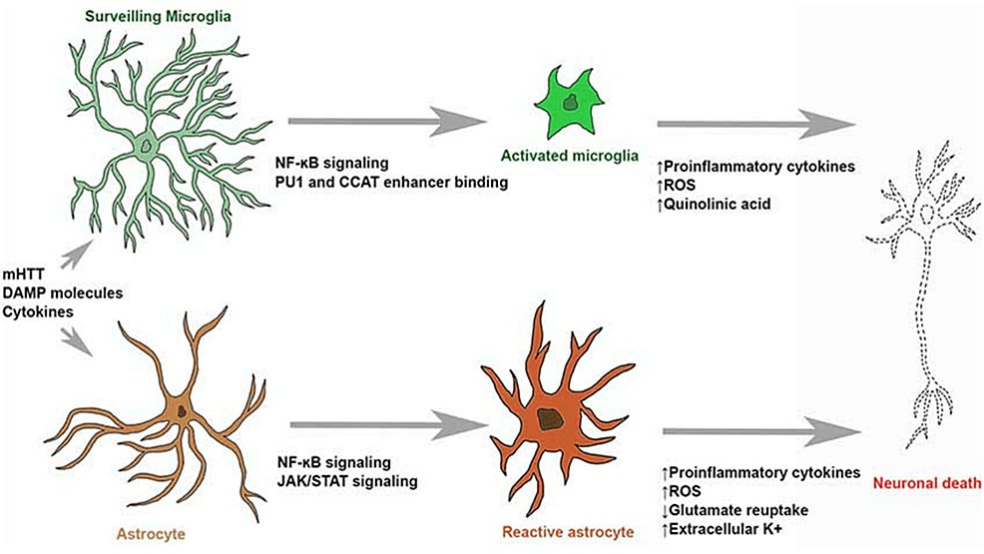
Image showing how microglial and astrocytic cells contribute to neuronal death in Huntington’s disease (HD). Surveilling microglia activate through NF-κB signaling, PU1 upregulation, and CCAT binding. Activated microglia and reactive astrocytes generate reactive oxygen species (ROS) and neurotoxic chemicals (such as quinolinic acid), which can initiate molecular processes that lead to neuronal death. Stimulatory molecules can also cause reactive astrogliosis, which raises pro-inflammatory cytokine levels, glutamate excitotoxicity, and neuron hyperexcitability. Reproduced under the terms of the Creative Commons Attribution License (CC BY) from Palpagama et al. [37]. Copyright © 2019 Palpagama, Waldvogel, Faull, and Kwakowsky.

Inflammation is the natural response to an infection or tissue damage, including protein misfolding. This response is mediated by cytokines and chemokines, which produce vasodilatation, swelling, and increased cell permeability, altering cell functionality when this becomes a chronic stimulus. Recently, studies have shown the role of neuroinflammation in the initiation, maintenance, and enhancement of dysfunctionality of neurons in the neurodegenerative process of diseases such as Alzheimer’s, Parkinson’s, epilepsy, stroke, Huntington’s, and ALS, among others. This study will review the suggested pathways of neuroinflammation [35]. When an infection strikes or tissue is damaged, especially by misfolding of proteins, inflammation is the normal reaction. When this becomes a persistent stimulus, the vasodilatation, swelling, and increased permeability caused by cytokines and chemokines that orchestrate this response change cell function. Neuroinflammation has been linked, in recent research, to the beginning, maintenance, and amplification of neuronal dysfunction in the neurodegenerative process of disorders such as Alzheimer’s, Parkinson’s, epilepsy, stroke, Huntington’s, and ALS [36]. This study will review the suggested pathways of neuroinflammation (Figure 3).

Apoptosis isms have been detected in both normal and Alzheimer’s brains. Apoptosis is triggered by two primary signaling pathways, namely, the intrinsic pathway regulated by mitochondria and the extrinsic pathway mediated by death receptors (DRs). The activation of pro-apoptotic caspases is required for signaling transmission once apoptosis has begun. Cyto c and APAF1 activate caspase-9, the first caspase in the intrinsic pathway, which then assembles apoptosomes. Caspase-8, the first caspase in the extrinsic route, is activated by the oligomerization of DRs and the subsequent recruitment of adaptor proteins, resulting in DISC. Activated caspases-9 and -8 then activate executioner caspase-3, resulting in neural cell death. Because apoptosis is poorly controlled in the brains of Alzheimer’s disease patients, both intrinsic and extrinsic pathways are activated. Abnormal protein aggregation and enhanced Fas ligands activate first caspases more effectively. The activation of early caspases leads to the activation of caspase-3, which results in a high rate of apoptosis during Alzheimer’s disease [37].

*Alzheimer*’s* Disease*

Inflammation is the body’s natural response to infection, damage to tissues, or misfolding of proteins. This response to chronic stimulation is facilitated by cytokines and chemokines, which increase cell permeability, edema, and vasodilatation.

Recent studies have demonstrated that neuroinflammation contributes to the beginning, maintenance, and amplification of neuronal dysfunction in the neurodegenerative process of diseases including Alzheimer’s, amyotrophic lateral sclerosis, Parkinson’s, stroke, Huntington’s, epilepsy, and many others. The suggested pathways of neuroinflammation will be investigated in this work [38].

Tau, a protein found in neurons, forms fibrillary intracytoplasmic complexes termed neurofibrillary tangles. One of the prominent roles of tau is to support axonal microtubules, which are required for transit inside the cell. Kinases add a phosphate group from ATP to tau proteins, which causes extracellular beta-amyloid to aggregate. Leading to neurofibrillary tangles, which are twisted paired helical filaments caused by tau aggregation [39]. Mislocalization and further protein misfolding result from this because it inhibits microtubule-dependent axonal transit. The fibrillar collagen found in these aggregates may trigger the release of cytokines and chemokines by microglial cells in the area. These secretions include IL-1B, IL-6, IL-12, IL-23, and TNF. These secretions facilitate vasodilation and permeability, stimulating the inflammatory pathway [40].

Microglia are immune-resident CNS macrophages that continuously patrol the CNS for damaged neurons, plaques, and infectious agents and react to minimize damage and inflammation. It has been suggested that they develop dual functions, either inducing inflammation and neurotoxicity or anti-inflammation and neuroprotection [41]. It is believed that they modify neuronal functioning when they get ramified and release cytokines and chemokines with immunological effects. A multifunctional cytokine that regulates the proliferation, activity, and survival of brain cells is TGF-β [42].

In addition, activated microglial cells can facilitate the production of various immune mediators by acting as antigen-presenting cells that stimulate and promote toll-like receptors. These cells, in turn, up-regulate the NF-κB, a nuclear transcription factor that activates genes involved in immune response, and the Janus kinase/signal transducers and activators of transcription pathway [43].

*Parkinson*’s* Disease*

Some people with Parkinson’s disease have impaired motor skills, cognitive abilities, emotional regulation, autonomic nervous system function, and even sensory abilities [44]. The substance nigra experiences a depletion of pigmented dopaminergic neurons. Dopamine increases cortical excitation via the direct pathway, which is mediated by D1 receptors, and inhibits the indirect pathway, which is mediated by D2 receptors. Unwanted movements result from an increase in the indirect pathway brought on by the loss of those neurons. Its pathology is via intracytoplasmic inclusions called Lewy body, which contains alpha-synuclein. Alpha-synuclein is a multimeric membrane-bound helical protein; half of it remains soluble as an unfolded monomer cytosolic protein, and has an N-Terminal region capable of interacting with the mitochondrial inner membrane component lipid cardiolipin, disrupting mitochondrial membrane integrity and leading to dysfunction. This mitochondrial inner membrane contains enzymes for electron transport and oxidative phosphorylation. Getting α-synuclein phosphorylation at Ser129 activates stress-signaling protein kinases and reactive species in microglia [45].

Because of its dense population of microglia, the substantia nigra is at increased risk of inflammatory assaults [46]. Overly activated microglia become more ameboid and undergo morphological changes, losing their beneficial function. An increase in inflammation is caused by the secretion of cytokines such as IL-1β, IL-2, IL-4, IL-6, TNF-α, TGF-α, and TGF-β1. It also causes vasodilation and increased cell permeability, which leads to damage to CNS tissues and the invasion of blood-borne inflammatory and immune cells. Kinases account for the majority of phosphotransferases [47]. They produce second messengers by moving a γ-phosphate group from ATP to various substances such as lipids, sugars, and proteins. Proliferation, differentiation, migration, and apoptosis are some of the subsequent processes that these molecules can initiate [48].

Through their interactions with lysosomes, endoplasmic reticulum, peroxisomes, and lipid droplets, mitochondria regulate signaling processes associated with autophagy and the immune response. They also play an essential role in ATP production, calcium balance, phospholipid transfer, and apoptosis [49]. When protein degradation decreases due to disruption, damaged misfolded proteins may be present.

As misfolded α-synuclein concentrations rise, it may increase demands for protein degradation and enhance proteolytic stress. It undergoes further pathological conformations as B sheet oligomers and amyloid fibrils and then the Lewy bodies, which are cytotoxic, eventually causing neuronal death after selectively reducing synaptic proteins, neuronal excitability, and neuronal networks. Furthermore, DA neurons are more susceptible to microglial activation and oxidative stress, and their antioxidant capacity is diminished due to mitochondrial organelle disruption, which may explain why intracellular glutathione levels in DA neurons are lower than in other regions of the substantia nigra [50].

Epilepsy

Neuroinflammation leads to glial cell activation and the secretion of inflammatory chemicals such as TNF-α, IL-1β, IL6, and HMGB1. By increasing permeability, lowering the integrity of tight junctions, and making metalloproteinases at the matrix more accessible, this inflammatory milieu undermines the BBB. A chronic epileptic focus and the etiopathogenesis of seizures are both impacted by this imbalance, which regulates the strength of transmission, affects neuronal plasticity, and modulates voltage- and receptor-gated ion channels. Some examples of DAMPs include the danger signal HMGB1, a highly motile non-histone nuclear protein, as well as other molecular patterns associated with danger, such as IL-1β activating IL-1R1, and TLR4. Protein kinases and gene transcription are involved in the cascade of events initiated by the cytokines, and NF-κB is essential for both processes [51]. Neuronal excitability and depolarization are enhanced via the transduction of signals related to proliferation, differentiation, motility, and adhesion when the NR2B subunit of the N-methyl-D-aspartate (NMDA) receptor is phosphorylated in response to IL-1R1. When TNF-α promotes the increased surface location of AMPA-type glutamate receptors, the neuron becomes more vulnerable to glutamate excitability and high intracellular calcium concentrations. A lower seizure threshold, cognitive deficits, and increased neuronal circuit hyperexcitability are the outcomes [52].

IL-6 is commonly recognized for its pro-inflammatory effects, particularly during the acute phase of inflammation where it promotes the production of other pro-inflammatory cytokines and recruits immune cells to the site of injury or infection. IL-6 also has significant anti-inflammatory functions. It promotes the production of anti-inflammatory molecules, such as IL-10 and soluble IL-1 receptor antagonists (sIL-1ra), which help mitigate the inflammatory response. Additionally, IL-6 can inhibit the production of TNF and IL-1, which are key drivers of inflammation. This dual role allows IL-6 to act as a regulator, balancing inflammation and promoting resolution once the initial inflammatory threat has been addressed [53].

AS enzyme detects cytosolic DNA, which can originate from pathogens or damaged host cells. Upon recognizing DNA, cGAS synthesizes a second messenger, cyclic GMP-AMP (cGAMP). cGAMP then binds to the STING receptor located on the endoplasmic reticulum membrane, triggering its activation. Once activated, STING initiates a signaling cascade that results in the production of type I interferons and other inflammatory cytokines. This enhances the immune response by promoting the activation of immune cells and the production of additional inflammatory mediators. The cGAS-STING pathway plays a critical role in detecting cellular damage and initiating appropriate inflammatory responses to maintain tissue homeostasis and protect against infections. Although primarily involved in initiating inflammation, the cGAS-STING pathway also contributes to the resolution phase by facilitating the clearance of cellular debris and promoting tissue repair. By balancing these functions, the pathway helps prevent chronic inflammation and maintains immune homeostasis. Normal brain function is disrupted, resulting in seizures, when the mechanisms that normally create a balance between inhibition and excitation break down. Process interference may cause seizures [54]. Blocking the sodium-potassium ATPase may cause seizures. There are several types of epilepsy, but TLE is particularly noteworthy. Seizures return after a while with no apparent cause in transitory encephalopathy. A characteristic of TLE pathogenesis, suggesting neuronal damage, is significant neuronal loss at postmortem [55]. Neuronal damage that cannot be repaired may result from abnormal changes in GABA, the primary neurotransmitter implicated in the disease of this homeostasis. Some of the most common reasons for seizures include infections of the brain, brain trauma, strokes, autism, dementia, and genetic disorders passed down through generations [56].

Stroke

Strokes may occur due to atherosclerosis in the brain’s blood vessels, embolisms, or the occlusion of small arteries. Recent findings reveal that neuroinflammation causes further damage, which ultimately causes cell death [57]. When neurons are damaged, the BBB breaks down, and immune cells such as T cells and neutrophils can enter the cerebral parenchyma. This triggers microglial cells to produce more cytokines, including nitric oxide, ROS, and TNF-α. The injured region grows as these cells acquire phagocytic and MHC class II antigen presentation capabilities, triggering further cell adhesion and leukocyte recruitment. Other effects of neuronal damage include excitotoxicity, Ca^2+^ influx, NMDA receptor overactivation, and glutamate release. Reducing glutathione levels after reperfusion is essential for astrocytes to prevent ROS-induced DNA damage. Astrocytes also emit ROS [58].

**Figure 4:**
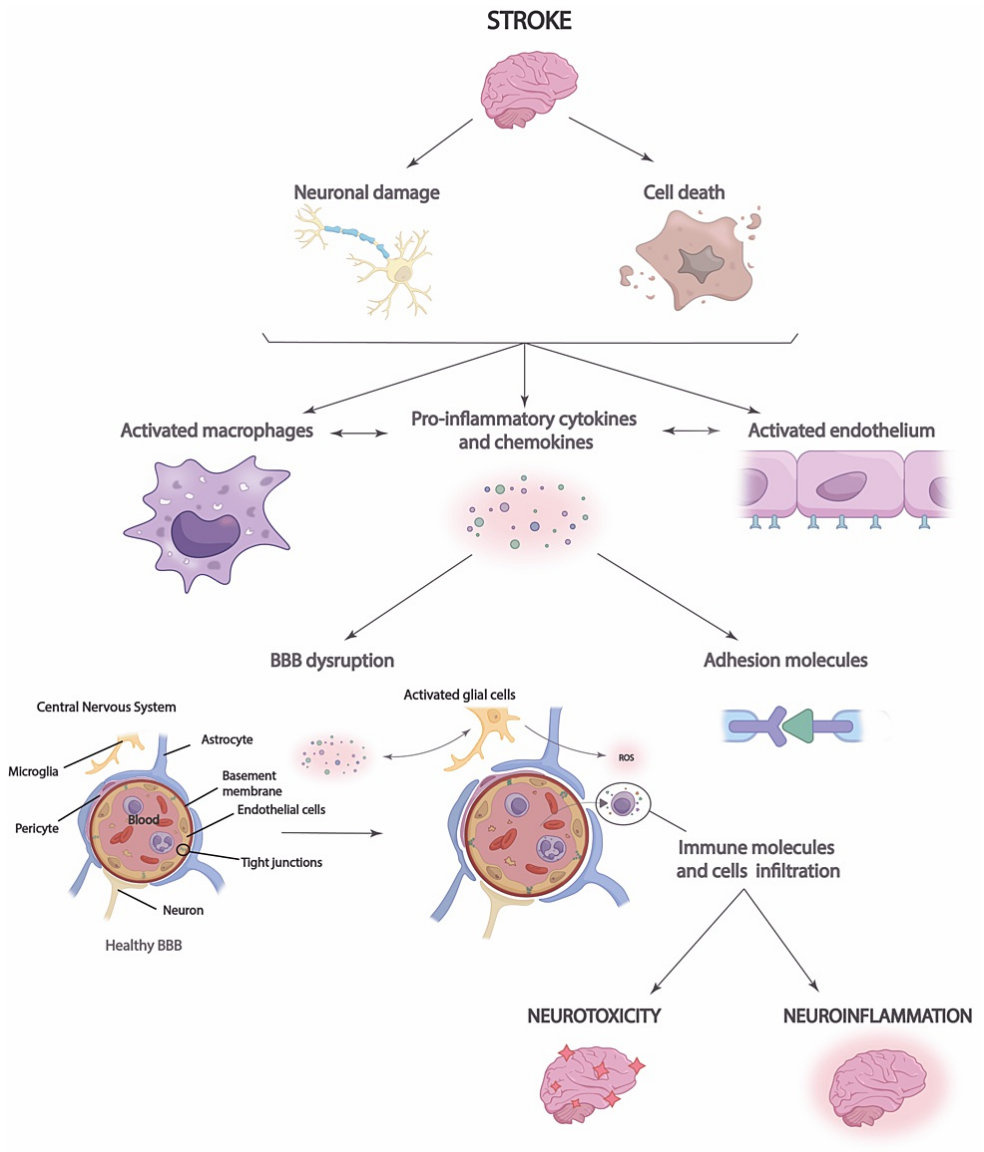
Stroke neuroinflammation process. The increased release of proinflammatory cytokines and chemokines following a stroke results in cell damage and neuronal death, which compromises the blood-brain barrier (BBB) and allows immune cells to infiltrate. This results in neuroinflammation and neurotoxicity in the brain. Reproduced under the terms and conditions of the Creative Commons Attribution (CC BY) license (https://creativecommons.org/licenses/by/4.0/) from Larrea et al. [60]. Copyright © 2023 by the authors. Licensee MDPI, Basel, Switzerland.

The most common kind of stroke is thrombosis, which leads to the acute deprivation of oxygen and glucose to brain tissue due to the blockage of a blood vessel. Among the several factors that contribute to the etiology of stroke are ischemic damage and inflammation. Permanent neuronal damage in the ischemic core is caused by the pathogenic pathways of the ischemic cascade minutes after cerebral ischemia starts. There are two main types of strokes, namely, acute ischemic stroke (85% of cases) and hemorrhagic stroke (15%). Ischemic strokes occur when blood flow to a region of the brain suddenly drops or stops altogether. Within seconds to minutes of a brain region losing blood flow, a series of events called the ischemia cascade starts, culminating in neuronal death and cell membrane permeabilization. A hallmark of oxidative stress, which occurs when the physiological balance between antioxidants and oxidants is disturbed, is ischemic cell death, which includes the production of reactive nitrogen species and ROS through various damage mechanisms, including mitochondrial inhibition (Figure 4) [59].

Late-Stage Amyotrophic Sclerosis

Paralysis and damage to the corticospinal tract and lower motor neurons in the brain and spinal cord are symptoms of a neurodegenerative disease that worsens over time. In ALS, more than 20 gene mutations have been identified, along with hexarepeat expansion on chromosome 9. ALS-related mutant proteins, such as the superoxide dismutase 1 gene and TAR DNA-binding protein-43, lead to oxidative damage [24].

Future directions and research

Several studies have been performed for a possible innovative drug delivery that could counteract the progression of Alzheimer’s disease. This method involves using phosphatidylserine nanoparticles complexed with microbubbles to oversee the inflammatory reaction. In a rat brain, this method was proven to successfully open the BBB and activate microglia and macrophages to allow rapid accumulation of nanoparticles of quercetin complexes that improved Parkinson’s disease regression [61]. There have been advancements in ultrasound contrast agents to enable products to move through the BBB to allow for the delivery of treatments such as gene therapy or drugs. Injecting microbubbles and the drug together while encapsulating them can allow for drug delivery across the BBB. Additionally, non-viral gene delivery could be accomplished by combining the necessary genes encoded in plasmid DNA and combining them with microbubbles to achieve delivery. It was successfully tested in a rodent model, and genes such as nuclear factor E2-related factor and brain-derived neurotrophic factor have been studied to be beneficial for the progression of Parkinson’s treatment. Precision medicine is when a person’s genetics, environment, and lifestyle are used to determine how to treat best or approach their disease. It is an emerging trend owing to advancements in gene understanding involving epilepsy. At the core of it, certain epilepsy conditions require a simple solution that gene therapy can provide, and precision medicine is used to facilitate this process. It is not a foolproof solution, as there have been instances when it did not work with certain conditions involving mutations, but nevertheless, it is a promising avenue being pursued [62]. Due to recent technological advancements, stroke mortality has never been lower, and new strategies have been implemented, such as telestroke and mobile stroke. New avenues have also been developed for post-stroke care healthcare such as treadmill exercise, electromechanical device therapy, and circuit class therapy [63].

Potential areas for future research

New therapeutic targets are being found every day that can have an impact on Alzheimer’s progression. Such targets include fenamate mefenamic acid, NLRP3 inhibitors, IL-1 receptor antagonists, neutralizing antibodies, MAPK alpha inhibitors, low-dose IL-2, C3aR antagonists, PD1 inhibitors, and CD33 inhibitors. All of these have been theorized in some way to help impact Alzheimer’s progression, and plans are in place for future studies involving them [64]. New strategies being developed involve transplantation of fetal tissue to replace lost DA neurons in Parkinson’s disease, converting stem cells from somatic cells into as many DA neurons as needed, clinical trials involving glial-derived neurotrophic factor, improving therapeutic delivery of such strategies like the stem cells, evolving current testing models, and using current advanced technology to bring together different species with humans for finding interspecies therapeutic advancements [65]. In the treatment of epilepsy, when medications fail, dietary therapies are popular, specifically involving the ketogenic diet. It is a popular therapy with promising results, but the question is when and who to prescribe it as opposed to starting treatment with medications. Newer diet therapies that have had successful results need to be further researched, such as the modified Atkins diet and low glycemic index treatment [66]. Genetic factors have a strong correlation with epilepsy, and it has become apparent that gene therapy is the future of epilepsy treatment. Medications and diet therapy have been successful and require more research, but they can have limitations compared to gene therapy. The main approach is gene transmission involving viruses as carriers encoding properties that, with more research, can bolster our understanding of epilepsy as a whole and treat it [67]. Many new developments are being researched to improve current stroke research. Looking toward the future, neuroprotective strategies, stem cell therapy, and involvement of the microbiota-gut-brain axis have shown promise [68].

Potential future treatments for neurodegenerative diseases

**Figure 5:**
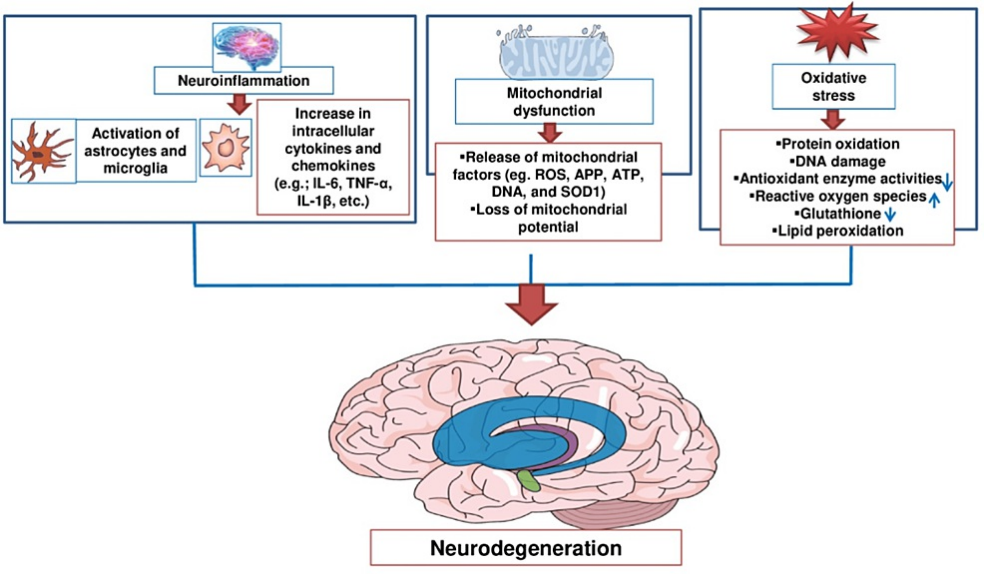
Therapeutic effects of numerous natural products for combating neurodegenerative diseases. Reproduced under the terms and conditions of the Creative Commons Attribution (CC BY) license (https://creativecommons.org/licenses/by/4.0/) from Rahman et al. [69]. Copyright © 2021 by the authors. Licensee MDPI, Basel, Switzerland.

Different and ever-evolving therapeutic methods are available for neurodegenerative diseases linked to neuroinflammation. Reducing disease progression, improving patient quality of life, and symptom management are the main objectives of these treatments. Natural products are also used to treat them (Figure 5).

Inflammatory Targets

The current pharmaceutical options only provide partial relief of symptoms, and no cures for neurodegenerative diseases are available. Still, a lot has been learned about how neuroinflammation plays a role in many diseases, and new medicines have emerged that target this critical pathway [70]. In a small group of individuals with mild-to-severe Alzheimer’s disease dementia, a recent phase II, double-blind, randomized study discovered that weekly subcutaneous injections of TNF-α (etanercept) had no significant effects when compared to a placebo. Important cues that activate the NLRP3 inflammasome include disturbances in molecules that modify homeostasis, patterns of molecules connected to pathogens, and patterns associated with danger. Several proteins, notably fibrillar β-amyloid, which are linked to diseases, may activate the NLRP3 inflammasome. In Alzheimer’s disease, this protein’s phagocytosis damages lysosomes, which release cathepsin B, activating the NLRP3 inflammasome [71].

Toll-Like Receptor

The innate immune system relies on toll-like receptors, vital for identifying patterns linked to infections or injuries and triggering immunological responses. They are present in immune and non-immune cells, where they cause inflammatory reactions by identifying specific ligands and initiating signaling pathways [72]. Their evolutionary preservation emphasizes their importance in the immune system. Several diseases have been associated with toll-like receptor dysfunction. By playing a role in neuroinflammation and neurodegeneration, TLR2 significantly affects neurodegenerative disorders. TLR2 has been shown to have a role in the onset and progression of Parkinson’s disease. The amount of TLR2 expression in neurons is directly correlated with the severity of the disease. Researchers found that TLR2 could contribute to disease advancement by preventing autophagy and promoting the accumulation of α-syn, a protein linked to the formation of Lewy bodies, a characteristic symptom of Parkinson’s disease [73]. Research on Parkinson’s disease models has revealed that mice deficient in the *TLR2* gene had reduced accumulation of α-synuclein and enhanced motor deficits. Recent research has shown that neuronal TLR2 may have a role in the dissemination of synucleinopathies as it is involved in the transmission of α-syn between neurons [74].

Antioxidants in Neurodegenerative Disease

The pathogenesis of Alzheimer’s disease is closely related to fundamental functional problems, such as mitochondrial failure and oxidative stress, which are hallmarks of Alzheimer’s disease. Researchers have recently conducted placebo-controlled clinical trials to examine the potential benefits of antioxidant-based treatments for individuals suffering from autoimmune disorders. The compounds administered and analyzed in this research include melatonin supplements, vitamin E, resveratrol, carotenoids, and omega-3 fatty acids. Several studies have shown that the non-flavonoid polyphenol resveratrol has neuroprotective, anti-inflammatory, and antioxidant effects. According to researchers, this substance successfully decreases the harmful effects and buildup of Aβ peptides in the hippocampus, a part of the brain linked to individuals with Alzheimer’s disease [75]. By decreasing the formation of Aβ1-42 in the hippocampus, resveratrol improves neurogenesis, protects the BBB, and stops the hippocampus from deteriorating.

According to a study, there is evidence that older adults may benefit from improved cognitive performance, particularly working memory, when they increase their intake of carotenoids, omega-3 fatty acids, and vitamin E. The effect of melatonin on sleep in Alzheimer’s disease patients has been the subject of clinical investigations. Results about changes in sleep habits have been rather noticeable. However, there is not much proof that it helps with cognition or health just yet, and that could be due to how short the treatments have been [76].

Nanoparticles in Neurodegenerative Disease

Nanomedicine is a medical field that combines nanotechnology to provide significant possibilities for detecting and treating diseases. Nanomedicine has a distinctive prospect of delivering pharmaceuticals specifically to targeted areas in the brain using drug-loaded nanoparticles [77]. Nanoparticles can envelop both hydrophilic and hydrophobic medicines, hence enhancing their solubility and stability. In addition, several inorganic or polymeric nanoparticles have been created that may effectively remove ROS and bind iron, making them a promising strategy for reducing iron overload-related damage and oxidative stress [78]. In addition, nanomedicine has shown potential in regulating both internal and extrinsic apoptotic pathways and decreasing the load of Aβ peptides, which play a crucial role in initiating Alzheimer’s disease. Exosomes, a form of nanoparticles, have been studied as a dependable approach in nanomedicine due to their capacity to cross biological barriers and reach tissues that lack blood supply [79]. Prior research has shown that exosomes generated from mesenchymal stem cells successfully restored the functionality of genes associated with synaptic plasticity and reduced the production of Aβ in individuals with Alzheimer’s disease. In addition, the findings showed that mice exposed to this therapy exhibited significant improvements in cognitive functions, reduced damage to neurons and astrocytes, and enhanced brain glucose metabolism. The present therapies for Alzheimer’s disease include nanostructure-based delivery methods, which may be classified into four primary categories, namely, metallic/non-metallic nanoparticles, organic nanoparticles, lipid vesicles, and emulsion-based systems [80].

## Conclusions

The role of neuroinflammation in degenerative diseases is prominent. Progress of neurodegenerative diseases directly depends on the release of inflammatory mediators. In the CNS, inflammatory effectors derived from innate and acquired immune systems, as well as glial cells, particularly microglia, act as sensors for disturbed brain tissue homeostasis and accumulate locally in response to neuronal cell injury or foreign entry in the brain. The differential activation of microglia cells is the central point that regulates neuroinflammation, which results in neurotoxicity or neuroprotection. Environmental exposure is, therefore, the critical element for the fate of neurons concerning degeneration or protection. Identifying key pathways and molecular targets associated with neuroinflammation will open new doors for potential therapeutic methods and interventions, even in severe debilitating conditions. Although inflammation is a natural immune response, the brain has a unique inflammatory response, allowing us to dive deep into this research. The immune response of the nervous system plays a role in the pathophysiology of epilepsy. Neural damage can trigger the cytokines to release and cause damage to neuron myelination, which can cause demyelinating diseases. BBB can get disrupted because of these distinctive inflammatory mediators by the brain’s immune system. The inflammatory reaction driven by the brain, followed by the necrosis and apoptosis due to stroke, is through the careful release of ROS, chemokines, and cytokines. The mediators from the immune cells cause neuronal impairment in degenerative disorders. This integrative approach is a new alternative road to studying these diseases. It is interesting to note in this context both genetic and environmental factors shape the immune system and the CNS. Many therapies targeted explicitly to different inflammatory signaling are in ongoing clinical trials, likely providing effective therapeutic methods for patients with neurodegenerative diseases in the future. A more complete understanding of the underlying cellular and molecular mechanisms of inflammation in neurodegeneration will be crucial to target inflammatory signals for the treatment of neurodegenerative diseases successfully.
